# The macro-economic determinants of health and health inequalities—umbrella review protocol

**DOI:** 10.1186/s13643-017-0616-2

**Published:** 2017-11-03

**Authors:** Yannish Naik, Peter Baker, Ian Walker, Taavi Tillmann, Kristin Bash, Darryl Quantz, Frances Hillier-Brown, Clare Bambra

**Affiliations:** 1 Leeds Teaching Hospitals NHS Trust, Leeds, UK; 2 Leeds Institute of Health Sciences (LIHS), Level 10, Worsley Building, Clarendon Way, Leeds, LS2 9NL UK; 3 Imperial College London, London, UK; 4 Nuffield Centre for International Health and Development, University of Leeds, Leeds, UK; 5 University College London, London, UK; 6 University of Lancaster, Lancaster, UK; 7 Institute for Health & Society, University of Newcastle, Newcastle upon Tyne, UK; 8 Newcastle University, Newcastle upon Tyne, UK

**Keywords:** Economics, Socio-economic determinants, Trade, Finance, Labour, Public-private, Market, Regulation, Production, Distribution

## Abstract

**Background:**

The economic determinants of health have been widely recognised as crucial factors affecting health; however, to date, no comprehensive review has been undertaken to summarise these factors and the ways in which they can influence health. We conceptualise the economy as a complex system made up of underlying approaches, regulation from institutions, markets, finance, labour, the public-private balance as well as production and distributional effects, which collectively impact on health through the effect of moderators. This protocol details the methods for an umbrella review to explore the macro-economic factors, strategies, policies and interventions that affect health outcomes and health inequalities.

**Methods:**

We will identify relevant systematic reviews using search terms derived from the *Journal of Economic Literatur*e classification. Reviews will be included if they meet the Database of Abstracts and Reviews of Effects criteria for systematic reviews. Reviews of studies with and without controls will be included; both association and intervention studies will be included. Primary outcomes will include but are not limited to morbidity, mortality, prevalence and incidence of conditions and life expectancy. Secondary outcomes will include health inequalities by gender, ethnicity or socio-economic status. Six databases will be searched using tailored versions of our piloted search strategy to locate relevant reviews. Data will be extracted using a standardized pro forma, and the findings will be synthesized into a conceptual framework to address our review aim.

**Discussion:**

Our umbrella review protocol provides a robust method to systematically appraise the evidence in this field, using new conceptual models derived specifically to address the study question. This will yield important information for policymakers, practitioners and researchers at the local, national and international level. It will also help set the future research agenda in this field and guide the development of interventions.

**Systematic review registration:**

This umbrella review protocol has been registered with PROSPERO CRD42017068357.

**Electronic supplementary material:**

The online version of this article (10.1186/s13643-017-0616-2) contains supplementary material, which is available to authorized users.

## Background

There has been long-standing recognition of the role of economic factors on health and well-being [1]. These economic factors are consistently identified in local, national and international population health frameworks as both influences on health and levers to improve health and reduce health inequalities. Building on these frameworks, there have been several efforts to quantify the impact of the social determinants relative to other influences such as healthcare services. In line with ongoing advocacy for investment in the wider determinants, the results indicate that social and economic factors are the largest influences on population health [2, 3]. In spite of this evidence and formal calls for action dating back to the Ottawa Charter, there continues to be frustration over the lack of practical policy interventions around social and economic determinants [4].

This lack of action has been attributed to the challenge of understanding the multifaceted impacts of social and economic factors, as well as the need for long-term outlooks to address policy problems [5, 6]. This is particularly pertinent in light of policy debates around Health in All Policies—a move to consider the impact on health and health inequalities in all aspects of government policy [7]. In response, there are calls to consider economic policies as health policies with research and evaluation that would more clearly identify the mechanisms through which economic factors/policies affect health [8]. Berkman notes that ‘understanding the ways in which social and economic policies impact population health is one of the most critical areas for public health today’ [9].

To date, public health researchers have begun to map out the interconnected pathways and linkages between economic conditions/policies and health outcomes. For example, the links between poverty and poor health are wide-ranging and well-documented with lower income being associated with both behavioural risk factors, such as smoking, and a range of negative health outcomes [10, 11]. Similarly, involuntary unemployment and its related income loss are associated with negative health outcomes [12].

More recently, there is growing media and public awareness over income inequalities which have continued to grow amongst OECD (Organization for Economic Co-operation and Development) countries over the last 30 years in spite of significant periods of economic growth [13, 14]. Wilkinson and Pickett have highlighted the body of evidence which shows the impact of income inequalities on a range of population health and social outcomes [15]. Recent research has also highlighted a potential link between ‘neo-liberal’ economic policies such as reductions in public sector spending and increased stress, obesity and health inequalities [16]. These growing income and wealth gaps, combined with environmental concerns, have instigated debate and proposals for alternative economic systems that could deliver improved health outcomes and reduced health inequalities [12, 15, 17].

Seminal social determinants reviews and commissions have begun to specify economic policies within recommendations frameworks. The 2008 *World Health Organisation Commission on Social Determinants of Health* (*SDH*) [18] listed a few of these elements, such as ‘social protection’, ‘progressive taxation’, ‘debt relief’ and ‘market responsibility’. This was further developed by the *European review of SDH* [19], which also looked at ‘gross domestic product (GDP), taxation and welfare’ and ‘economics’. These prominent reports have drawn upon the wide literature of associations between health and, as Dahlgren and Whitehead [20] described it, the general socio-economic, cultural and environmental conditions in society, and also began the process of formalizing these into core themes and categories. The overall pattern is one where purely economic determinants are frequently conceptualized alongside other wider determinants of health (such as environmental and political determinants). This frame might have been too broad, thereby preventing the formation of a comprehensively structured schema of the Economic Determinants of Health. On the other hand, we recognize recent work to define narrower constructs, such as the commercial determinants of health [21]. In our view, these could nonetheless be nested with a wider framework of Economic Determinants of Health, which we hope our review could inform.

The literature has now gone beyond investigating the determinants to identifying potential interventions. Khan et al. carried out a rapid scoping review [22] and found 195 systematic reviews of economic interventions. They found that taxes and subsidies could be used to encourage use of services and healthy patterns of consumption, income transfer programs can support individuals to meet their needs and encourage treatment adherence and that incentives can be used to alter provider and patient behavior. They also found that livelihood support programs can help to increase income, through supporting people to earn a higher income for example. Finally, they found that health-related financial services such as insurance could help householders to manage healthcare-related financial risk. However, we suggest that their definition of economic interventions is limited, ignoring, for example, macro-economic interventions such as fiscal policy and trade policy. Despite progress to date, no comprehensive overview of the macro-economic determinants of health, their relative importance and the different mechanisms through which they affect health has been produced.

We thus aim to carry out a review to provide a rigorous evidence base around the macro-economic determinants of health and health inequalities. We aim to provide a conceptual model to understand the links between the economy and health and use this conceptual model to explore the existing evidence base systematically. We will thus provide evidence to policymakers, researchers and health advocates which can be used to develop evidence-based economic policy interventions and clarify priorities for further research. Given the broad scope of this research question and the large number of existing systematic reviews on each of its subtopics, we aim to carry out an umbrella review—a methodology which involves carrying out a systematic review of reviews [23].

The economy has been defined as a ‘social domain that emphasizes the practices, discourses, and material expressions associated with the production, use and management of resources.’ [8]. The economy is thus conceived of as a complex interacting system which influences health through a number of mediators (access to healthcare, housing, etc.).

**Fig. 1:**
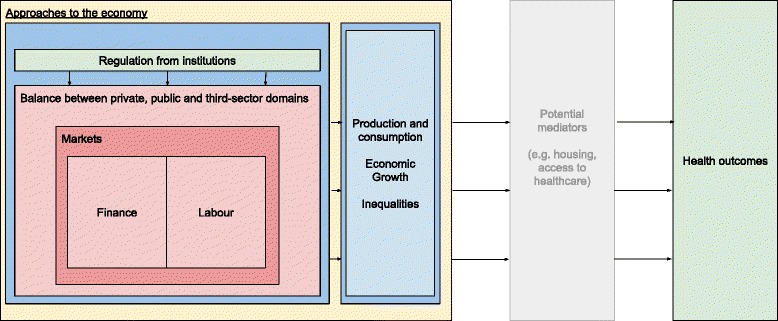
Conceptual model of links between the economy and human health

The *Journal of Economic Literature* (JEL) provides a classification [24] of the key concepts that relate to research in economics. Based on the JEL terms and our conceptual framework, we propose that the economic factors that influence health can broadly be conceived of in seven major categories—market regulation; institutions; supply of money; finance and loans; the balance between the public, private and third sector; labour; production and consumption and approaches to the economy. Table 1 presents these seven categories, related subtopics for each category at the local, national and international level as well as illustrative examples of potential health implications. Whilst we acknowledge that this list is not exhaustive, it provides an initial framework to guide our search strategy. We also propose an a priori simplified framework (Fig. 1) to show the broad relationships between economic factors and health that we are investigating.

**Table 1:** Matrix of economic factors at local, national and international level

	Local level	National	International	Illustrative example of impact on health (if known)
Category 1: market regulation		Competition including legislation, consideration of externalities in pricing, fiscal measures, e.g. tax, market structure	Trade policy	Regulation of the tobacco market, via taxation and restrictions on advertisement and right to trade with tobacco has been associated with a range of benefits such as reduced heart disease [25]
Category 2: institutions		Central bank, banks, micro-finance, mortgages, startups. Legislation and regulation of organisations	International organisations, e.g. International Monetary Fund, World Bank, multinational firms, World Trade Organisation	Loans issued by the IMF and subsequent tuberculosis mortality [26]
Category 3: supply of money, finance and loans	Local currencies, debt	Interest rates, inflation, deflation, wages, supply of money or credit, macro-economic policy, fiscal policy, financial crises, monetary policy, structural adjustment policies, natural resources	International lending, foreign aid, financial transactions tax, capital controls	Financial crises and suicide rates [27]
Category 4: balance between public, private and third sector	Land tenure Informal economies, shadow economies, social enterprises and cooperatives	Structure and scope of government, privatization and nationalization, taxation, tax avoidance, government expenditure and welfare provision, property rights		Mass privatization and mortality in the former Soviet Union [28]
Category 5: labour	Firm governance, structure, ownership, behavior,	Trade unions, employment, unemployment, minimum wage, labor force size and structure		Unemployment and suicide [29] OR Overwork and stroke [30]
Category 6: production and consumption	Income, wealth, distribution	Industrialisation, economic growth and aggregate productivity		Income inequality and mortality [31]
Category 7: approaches to economy	Regional economics	Capitalist, socialist, transitional, Keynesian, Marxian, neoclassical, ecological economics		Political traditions more committed to redistributive economic policies may lead to improvements in the health of populations [32]

Some factors could be in multiple categories. They have been assigned to the most relevant category

## Methods

We used the Preferred Reporting Items for Systematic review and Meta-Analysis Protocols **(**PRISMA-P) checklist [33] to develop this umbrella review (see Additional file 1 for the completed checklist).

### Research question

What are the macro-economic factors, strategies, policies and interventions that affect health outcomes and health inequalities?

### Study design

The umbrella review method provides a transparent and rigorous approach to reviewing the highest quality evidence on a broad topic and is increasingly common in the field of public health [34–37]. The protocol for this systematic review is registered on PROSPERO (CRD42017068357), and any amendments will also be registered.

### Inclusion criteria

Two restrictions of our scope are important to state. Firstly, the economy is usually analyzed on two levels: the micro level—concerned with economic decision-making at the individual and household level—and the macro level—concerned with large-scale aspects such as the size of the economy, monetary policy, labour policy and trade. This review will focus on population-level macro-economic determinants and associated health outcomes, because there has already been substantial work on micro-economics and health [38, 39]. However, we recognize that these distinctions may be contentious, and thus we have had to use our judgment to draw pragmatic boundaries around the scope of the study.

Secondly, economic factors impact on health partly through mediators such as healthcare expenditure, other social determinants of health such as housing and environmental factors such as pollution and climate change. Each of these mediators is likely to involve a complex causal chain with a significant evidence base around its health impacts. It is not practically possible to review the impact of the economy on each of these mediators or the impact of each of these mediators on health as each of these would likely require a separate systematic review. We will thus not include these mediators within our review. Instead, we will focus on reviewing the overall association between economic factors and health. The inclusion criteria for our review of systematic reviews are listed in Table 2.

**Table 2:** Criteria for including systematic review articles, in the present umbrella review

Study design	Systematic reviews meeting Database of Abstracts of Reviews of Effects (DARE) [40] criteria: (i) a defined review question (which includes at least two out of population, intervention, comparison, outcomes or study designs), and with a search strategy of a named database, and (ii) a search strategy including both a named database (at least) and one of the following: reference checking, hand searching, citation searching or contact with authors. These reviews can include observational and experimental studies—for example randomised and non-randomised studies, cohort studies, intervention studies and cross-sectional association studies, as well as effectiveness, cost-effectiveness, modelling and implementation studies.
Timeframe	No restriction based on the length of follow-up of outcomes.
Population	Adults and children in high-, low- and middle-income countries.
Intervention/exposure	The reviews must primarily focus on macro-, population-level rather than individual-level economic determinants of health. This may include reviews of association and modelling studies as well as reviews of intervention studies.
Comparator	Systematic reviews of studies with and without controls will be included
Outcome	Health and health inequality outcomes. Primary outcomes including but not limited to morbidity, mortality, prevalence and incidence of conditions and life expectancy. Secondary outcomes include health inequalities by gender, ethnicity or socio-economic status (for example by income, education, employment, receipt of benefits at an individual or area level). Cost-effectiveness data will also be extracted if available.
Setting	Any setting—low, middle, high-income countries.
Year considered	All years since the start of database.
Language	English language
Publication status	Only peer-reviewed published studies

### Search strategy

We will search Medline (Ovid), Embase (Ovid), Econlit (EBSCO), PsycInfo (Ovid), Applied Social Sciences Index and Abstracts (ASSIA; ProQuest) and Sociological Abstracts (ProQuest) for relevant papers, developing tailored searches for each database. This will ensure we will synthesize the best evidence from all the relevant disciplines. We will hand-search key reviews for further citations. We will only include peer-reviewed published studies and so will exclude grey literature to ensure the highest quality of evidence is reported.

### Screening, data extraction and quality appraisal

Core members of the review team will screen the titles and abstracts to exclude irrelevant papers, with a second author screening a random 10% sample [37]. A lead author will act as arbitrator in case of disagreement. Two authors will independently review the full text of articles to determine inclusion, discussing any disagreement and, if need be, discussing this with a third author also. We will calculate the percentage agreement between reviewers at the title and abstract screening stage and for full-text inclusion.

We will extract key data from full-text versions of included papers using standard extraction forms adapted from previous reviews for this purpose [34, 37] (see Table 3 for key data points to be extracted). We will then quality appraise the reviews selected using the Assessment of Multiple Systematic Reviews (AMSTAR) approach [41] as part of our standard extraction form. This approach explores for example study selection and extraction, search details, methods of synthesis, assessment of publication bias and conflict of interest. It is now widely accepted as part of umbrella review methodology being easy to use and having been externally validated [42, 43].

**Table 3:** Data extraction fields

Review characteristics	Results of review
• Key economic determinants identified and their conceptual synonyms • Economic characteristic, strategy, policy or intervention? • Outcomes used • Population • Setting • Number of studies included in review • Number of databases searched and disciplines • Was grey literature searched or citation follow-up carried out? • Types of studies included • Synthesis methods—narrative/meta-analysis/realist/etc. • Time/language restriction	• Main results and strength of findings including variations by gender, ethnicity or socio-economic status • Quality of underlying evidence: risk of bias and confounding, consistency across multiple settings. • Proposed mechanistic pathways • Clear evidence gaps identified • Key contextual factors (e.g. political/social/historical)

### Synthesis

Where a meta-analysis has been carried out, we will report the combined effect size. Where the review does not provide a summary measure of effect, we will explore the key findings and use these to inform a narrative overview of the key findings. We will also discuss the methodological weaknesses of the studies underlying them [37] and offer recommendations on future study designs. We do not plan to carry out meta-analysis given the broad topic being studied.

Through this synthesis, we aim to summarise the latest evidence in this field and develop a conceptual framework that will provide insight into the different subtopics under study. The findings of our review could be applied as recommendations for practice for a range of stakeholders, as well as helping to identify major gaps in the evidence to set future research agendas.

## Pilot search strategy

We developed an ‘economic’ search strategy from the American *Journal of Economic Literature* (JEL) classification system [24] which is used to categorise economic literature (see Additional file 2 for JEL terms that were included and excluded). We included JEL terms focused on the economy or its key components or policies (see Table 1) and excluded JEL categories that were purely theoretical or methodological, or that were focused on the micro level (individual or household).

We then developed a ‘health outcomes’ search based on key concepts which might be associated with the economic terms we chose, and on a previously used search [36]. In line with this previous umbrella review, we decided not to include specific inequality terms as these might excessively restrict our search results. We modified the Scottish Intercollegiate Guidelines Network (SIGN) terms to limit our search to systematic reviews [44], ensuring the search was more specific than previously used searches [45] for pragmatic reasons.

We refined search terms through consultation with topic experts and information specialists. We further developed the searches through an iterative process, including Medical Subject Headings (MeSH terms) as appropriate [46]. This involved a number of pilot searches followed by a discussion of possible amendments to our strategy at each stage and refinement.

Given the breadth of the review, it was important to balance the sensitivity and specificity of our search strategy. To do this, we identified terms that might have ambiguous health and economic concepts (e.g. deprivation) which would have reduced the specificity. We re-specified or removed them as appropriate to ensure the search remained focused. We developed a pilot search strategy for Medline (see Table 4 for number of results). We tested the sensitivity of the pilot search strategy by checking the inclusion of key ‘tracer papers’—these are papers we would expect our search to find, in line with previous umbrella reviews [35–37] (see Table 4). All tracer papers were picked up by the pilot search; therefore, the search strategy was finalised for Medline (Additional file 3) and will be adapted for other databases.

**Table 4:** Pilot search strategy using Medline via Ovid, from start to present date including new and in process (searched on 6/6/2017)

	Search 1: health terms	Search 2: combine health terms with economics terms	Search 3: limiting search 2 to systematic reviews
Source of key words (see Additional file 3 for full list)	Adapted from a previous umbrella review [37]	Developed from JEL [24]	Modified SIGN systematic review filter [44]
Results	7,211,084	359,898	7087
Tracer papers			
Roelfs et al. [47]	Y	Y	Y
Parmar et al. [48]	Y	Y	Y
Roy et al. [49]	Y	Y	Y
Iemmi et al. [50]	Y	Y	Y

## Discussion

This umbrella review will provide, for the first time, a systematic overview of economic determinants of health. It will offer a broad overview of existing evidence and identify key gaps in the current knowledge. We will seek to use the results in a novel conceptual framework which will assist in bringing together the diverse disciplines that inform this field. This can be used to inform international, national and local policy to improve health. Understanding the impact that macro-economic determinants have on health, and gaps in this evidence, will also help set the future research agenda in this field and guide the development of interventions. Building on previous reviews, we will also discuss the impact of context on the economic determinants of health [37] given the increasing recognition of the importance of contextual factors in public health [51].

## Additional files

Additional file 1: PRISMA-P checklist. (DOC 84 kb)

Additional file 2: AEA JEL codes for inclusion. (DOCX 85 kb)

Additional file 3: Search strategy for Medline. (DOCX 94 kb)

## Abbreviations

AMSTAR: Assessing the Methodological Quality of Systematic Reviews
DARE: Database of Abstracts and Reviews of Effects
GDP: Gross domestic product
JEL: Journal of Economic Literature
MeSH: Medical Subject Headings
OECD: Organization for Economic Co-operation and Development
PRISMA-P: Preferred Reporting Items for Systematic review and Meta-Analysis Protocols
PROSPERO: International Prospective Register of Systematic Reviews
SDH: Social determinants of health
SIGN: Scottish Intercollegiate Guidelines Network
