# Correction to: The macro-economic determinants of health and health inequalities—umbrella review protocol

**DOI:** 10.1186/s13643-019-1148-8

**Published:** 2019-09-07

**Authors:** Yannish Naik, Peter Baker, Ian Walker, Taavi Tillmann, Kristin Bash, Darryl Quantz, Frances Hillier-Brown, Clare Bambra

**Affiliations:** 1 Leeds Teaching Hospitals NHS Trust, Leeds, UK; 2 Leeds Institute of Health Sciences (LIHS), Level 10, Worsley Building, Clarendon Way, Leeds, LS2 9NL UK; 3 Imperial College London, London, UK; 4 Nuffield Centre for International Health and Development, University of Leeds, Leeds, UK; 5 University College London, London, UK; 6 University of Lancaster, Lancaster, UK; 7 Institute for Health & Society, University of Newcastle, Newcastle upon Tyne, UK; 8 Newcastle University, Newcastle upon Tyne, UK

**Correction to: Syst Rev (2017) 6:222**

**https://doi.org/10.1186/s13643-017-0616-2**

Following publication of the original article [1], the authors opted to correct the following reference on page 3:

- The economy has been defined as a ‘social domain that emphasizes the practices, discourses, and material expressions associated with the production, use and management of resources’ [8].

It should have been:

- The economy has been defined as a ‘social domain that emphasizes the practices, discourses, and material expressions associated with the production, use and management of resources’ [52].

52. James P, Magee L, Scerri A, Steger MB (2015). Urban Sustainability in Theory and Practice: Circles of Sustainability. London: Routledge. p. 53.

The authors apologize for the inconvenience caused.
