# Supplementary material for: The macro-economic determinants of health and health inequalities—umbrella review protocol
Source: Syst Rev. 2017 Nov 3;6:222. doi: 10.1186/s13643-017-0616-2 (PMC5670527; doi:10.1186/s13643-017-0616-2)
Supplement: Supplementary file 2 — AEA JEL codes for inclusion. (DOCX 85 kb) [file 13643_2017_616_MOESM2_ESM.docx]

**Additional file 2 - AEA JEL codes for inclusion**

(<https://www.aeaweb.org/econlit/jelCodes.php?view=jel#E>)

|  | Highest level JEL term Included for searching Econlit | Words from JEL subcategories for searching other databases | JEL categories excluded and reason for exclusion |
| --- | --- | --- | --- |
|  |  |  | A. General Economics and Teaching - not relevant - just theory  B History of Economic Thought - not relevant - just theory  C Mathematical and Quantitative Methods - not relevant - just methodological |
| D | D02 Institutions: Design, Formation, Operations, and Impact  D04 Microeconomic Policy: Formulation, Implementation, and Evaluation  D2 Production and Organizations  D3 Distribution  D4 Market Structure, Pricing, and Design  D62 Externalities  D63 Equity, Justice, Inequality, and Other Normative Criteria and Measurement | D21 Firm Behavior  D23 Property Rights  D24 Productivity  D31 Personal Income, Wealth, and Their Distributions  D41 Perfect Competition  D42 Monopoly  D43 Oligopoly | D01 Microeconomic Behavior: Underlying Principles  D02 Institutions: Design, Formation, Operations, and Impact  D03 Behavioral Microeconomics: Underlying Principles   - 3 not relevant - just theory   D1 Household Behavior and Family Economics - Not revelant - not focused on economy  D5 General Equilibrium and Disequilibrium -not relevent - just theory  D61 Allocative Efficiency • Cost–Benefit Analysis - excluded to avoid large and irrelevant cost-effectiveness literature  D64 Altruism • Philanthropy • Intergenerational Transfers† - not relevant  D7 Analysis of Collective Decision-Making - not relevant  D8 Information, Knowledge, and Uncertainty - Not relevant  D9 Intertemporal Choice - not relevant |
| E | Whole section - Macroeconomics and Monetary Economics | E1 General Aggregative Models  E11 Marxian  E12 Keynesian  E13 Neoclassical  E2 Consumption, Saving, Production, Investment, Labor Markets, and Informal Economy  E24 Employment • Unemployment • Wages • Intergenerational Income Distribution • Aggregate Human Capital • Aggregate Labor Productivity  E31 Price Level • Inflation • Deflation  E32 Business Fluctuations • Cycles  E4 Money and Interest Rates  E42 Monetary Systems  E44 Financial Markets  E5 Monetary Policy, Central Banking, and the Supply of Money and Credit  E6 Macroeconomic Policy  E62 Fiscal Policy |  |
| F | F01 Global Outlook  F02 International Economic Order and Integration  F1 Trade  F2 International Factor Movements and International Business  F3 International Finance  F4 Macroeconomic Aspects of International Trade and Finance  F6 Economic Impacts of Globalization | F13 Trade Policy • International Trade Organizations  F15 Economic Integration  F23 Multinational Firms  F24 Remittances  F33 International Monetary Arrangements and Institutions  F34 International Lending and Debt Problems  F35 Foreign Aid  F38 International Financial Policy: Financial Transactions Tax; Capital Controls  F41 Open Economy  F44 International Business Cycles | F5 International Relations, National Security, and International Political Economy - not relevant to economy |
| G | G01 Financial Crises  G1 General Financial Markets  G2 Financial Institutions and Services  G3 Corporate Finance and Governance | G21 Banks • Depository Institutions • Micro Finance Institutions • Mortgages  G31 Financial Policy | G02 Behavioral Finance: Underlying Principles - not relevant, theory only |
| H | Whole section - Public Economics | H1 Structure and Scope of Government  H13 Nationalization  H2 Taxation, Subsidies, and Revenue  H23 Externalities • Redistributive Effects •  H26 Tax Evasion and Avoidance  H41 Public Goods  H5 National Government Expenditures  H53 Government Expenditures and Welfare Programs  H54 Public Investment  H55 Social Security and Public Pensions  H6 National (and local) Budget, Deficit, and Debt |  |
| I | I10 Health - General  I14 Health and Inequality  I15 Health and Economic Development  I18 Government Policy • Regulation • Public Health  I19 Health - Other  I31 General Welfare, Well-Being  I38 Government Policy • Provision and Effects of Welfare Programs |  | I11 Analysis of Health Care Markets  I12 Health Behavior  I13 Health Insurance, Public and Private   - the above 3 excluded to as do not focus on the economy and its link to health   I2 Education and Research Institutions - not relevant to economy  I32 Measurement and Analysis of Poverty - not relevant - methodological |
| J | J08 Labor Economics Policies  J2 Demand and Supply of Labor  J3 Wages, Compensation, and Labor Costs  J4 Particular Labor Markets  J5 Labor–Management Relations, Trade Unions, and Collective Bargaining  J6 Mobility, Unemployment, Vacancies, and Immigrant Workers  J7 Labor Discrimination  J8 Labor Standards: National and International | J21 Labor Force and Employment, Size, and Structure  J24 Human Capital • Labor Productivity  J51 Trade Unions  J61 Geographic Labor Mobility • Immigrant Workers  J64 Unemployment  J65 Unemployment Insurance  J8 Labor Standards:  J83 Workers' Rights | J00 General - not relevant - theory only  J01 Labor Economics: General - not relevant - theory only  J1 Demographic Economics - not relevant to the economy’s impact on health |
|  |  |  | K - Law and Economics - not relevant to the impact of the economy on health |
| L | Whole section - Industrial Organization | L1 Market Structure, Firm Strateg  L12 Monopoly  L13 Oligopoly  L15 … Industrial Structure  L2 Firm Objectives, Organization, and Behavior  L3 Nonprofit Organizations  L32 Public Enterprises • Public-Private Enterprises  L33 Privatization  L44 Antitrust Policy  L52 Industrial Policy |  |
| **M** | M13 New Firms • Startups  M2 Business Economics  M3 Marketing and Advertising  M38 Government Policy and Regulation |  | Rest of M1 Business Administration - too focused on HR, not relevant to economy  M4 Accounting and Auditing - not focused on the economy  M5 Personnel Economics - not focused on the economy  M14 Corporate Culture • Diversity • Social Responsibility – not focused on economy |
|  |  |  | N. Economic History - not focused on contemporary links between economy and health |
| O | Whole section - Economic Development, Innovation, Technological Change, and Growth | O14 Industrialization • Manufacturing  O16 Financial Markets  O17 Formal and Informal Sectors • Shadow Economy  O18 Urban, Rural, Regional,  **O**4 Economic Growth and Aggregate Productivity | O13 Agriculture • Natural Resources • Energy • Environment • Other Primary Products  “Technological Change” |
| P | Whole section - Economic Systems | P12 Capitalist Enterprises  P13 Cooperative Enterprises  P14 Property Rights  P16 Political Economy  P2 Socialist Systems and Transitional Economies |  |
| Q | Agricultural and Natural Resource Economics; Environmental and Ecological Economics | Q15 Land Ownership and Tenure • Land Reform    Q57 Ecological Economics: Ecosystem Services • Industrial Ecology | Q52 Pollution  Q53 Hazardous Waste • Solid Waste • Recycling Q54 Climate • Natural Disasters • Global Warming  Q56 Sustainability • Population Growth  Q2 Renewable Resources and Conservation  Q33 Resource Booms  Q34 Natural Resources  Q35 Hydrocarbon Resources  Biodiversity  Conservation |
| R | R1 General Regional Economics  R5 Regional Government Analysis |  | R2 Household Analysis - not focused on the broad economy  R3 Real Estate Markets, Spatial Production Analysis, and Firm Location - to specific, not focused on the economy  R4 Transportation Economics - to specific, not focused on the economy |
|  |  |  | Y. Miscellaneous Categories and Z. Other Special Topics - both too specific and not relevant |
